# Adolescents’ health-related quality of life (HRQoL) changes over time: a three year longitudinal study

**DOI:** 10.1186/s12955-016-0415-9

**Published:** 2016-01-25

**Authors:** Tanya Meade, Elizabeth Dowswell

**Affiliations:** School of Social Sciences and Psychology, Western Sydney University, Locked Bag 1797, Penrith, NSW 2751 Australia

**Keywords:** HRQoL, KIDSCREEN, Australian adolescents, Longitudinal study, Physical health, Psychological health, Mental health

## Abstract

**Background:**

Adolescence is a significant developmental stage marked by physical, psychological and social changes. While adolescents are generally perceived to be healthy, this stage of development is also associated with an emergence of risk factors that may have long-term consequences for their wellbeing. The aim of this study was to assess health related quality of life (HRQoL), and possible gender and age differences, in a sample of secondary school-aged adolescents over a three-year time period.

**Methods:**

Australian adolescents (*n* = 403, aged 12–15 at baseline) across six New South Wales high schools completed the KIDSCREEN-27 Questionnaire at three time points. The KIDSCREEN-27 measures five HRQoL domains (physical wellbeing, psychological wellbeing, autonomy and parents relations, social support and peers, and school environment). Mixed-between-within-subjects ANOVA analyses were employed to examine HRQoL over time and across age and gender.

**Results:**

HRQoL rates were comparable to the European-based KIDSCREEN norms with the exception of psychological wellbeing, which was considerably lower in this study’s sample.

Over time, for the total sample, there were significant changes on only one of the five dimensions (social support and peers). However, gender differences were found to be significant across three dimensions (physical wellbeing, psychological wellbeing, and autonomy and parents relations), with females reporting lower scores than males (i.e. lower HRQoL). Females’ scores also declined over the three time points across two of the five HRQoL dimensions (social support and peers, and school environment), indicating reductions in HRQoL over time. Age differences were found across all but one dimension (autonomy and parents relations).

**Conclusions:**

Although statistically significant, the changes in HRQoL may not be clinically significant, as the effect sizes were small and therefore those changes would not be readily noticeable. Those changes, however, suggest that, while HRQoL is predominantly stable over time, fluctuations and declines, such as those found for females, may be early indicators of physical and psychological vulnerabilities. If such vulnerabilities are detected timely; they may be addressed with preventative measures or appropriate interventions.

## Background

Adolescents are generally perceived to be healthy [1] and yet a systematic analysis of premature mortality and years lost due to disability indicates that young people (aged 10–24) who represent around 27 % of the global population account for up to 15 % of global disease burden [2]. The highest risk factors include neuropsychiatric disorders (45 %), unintentional self-harm (12 %), and infectious and parasitic diseases (10 %) [2]. While risk factors and lifestyle choices such as alcohol consumption may not have an immediate effect, they can be associated with negative long-term consequences [3, 4].

Psychological/mental health problems, which may emerge during this developmental stage [5], can often contribute to, or co-exist with physical health concerns, and persist into adulthood i.e. [6, 7]. These psychological and physical health problems, or vulnerabilities, can form an indivisible comorbidity [8], which can lead to poorer quality of life in adulthood compared to those that may not have concurrent health concerns [9]. Mental health problems in adolescents are often related to risky behaviours (i.e. substance abuse and unsafe sex) [10] and comorbid physical health problems include obesity, diabetes, cardiovascular disease, and HIV [11]. For example, a two-year longitudinal study (*n* = 1,332, age range = 9–18, 33 % female), found mood disorders to be associated with infectious disease and respiratory and weight problems, while disruptive behaviour disorders were related to higher risk-related behaviours (i.e. sexually transmitted diseases, drug overdose, suicide attempt) [12].

While adolescents have the highest prevalence of mental health problems across all age groups, less than a quarter of them access relevant health services [13–16]. These mental health problems, while not necessarily clinically significant at the time, may be predictive of broader health concerns in the future [8]. It is therefore important to observe adolescents’ health (i.e. psychological, physical, social) over time to detect early indicators of decline and provide prevention or intervention, if necessary, to inhibit the development of long-term consequences [3]. Furthermore, given that adolescents spend a great deal of time in school, the educational setting presents an optimal environment to assess adolescents’ wellbeing and, if necessary, facilitate pathways into services.

Health related quality of life (HRQoL) is a concept inclusive of mental, physical, and social wellbeing. Measuring HRQoL is increasingly seen as a useful indicator of health outcomes and health services effectiveness [17, 18], although yet to be fully utilised in child and adolescent population [18]. One instrument specifically developed to evaluate children and adolescents’ HRQoL, is the KIDSCREEN questionnaire [19]. While it is not specifically designed to measure health outcomes, it provides a comprehensive indication of overall health/wellbeing with age-based norms. The KIDSCREEN questionnaires were developed based on a data collected across 13 European countries to establish age-range norms for eight to 18 years olds [20], and a series of studies were conducted to examine age, gender and socioeconomic inequalities in quality of life [21]. In the absence of significant health threats or life events, HRQoL is considered to be generally stable across adolescents [22, 23], although some studies have found HRQoL to decline during adolescence [24–26]. Physical and mental well-being in particular seem to deteriorate with age and more so for females than males. These changes may be due to the developmental challenges that adolescent’s experience characterised by physical, psychological, vocational and social changes, while gender differences may be due to contradictory social demands placed on girls [24] and subsequent vulnerability to physical and psychological health challenges [27]. Similar age and gender differences have been reported across Australian populations including adolescents on remand and adolescents with excess weight [28, 29] using other quality of life measures.

Of the studies that have used KIDSCREEN instruments, only six have been longitudinal, ranging from 12 months to five years follow-up [22, 23, 30–33]. However, four of these studies were conducted with specific populations, including adolescents with cerebral palsy [30], obesity [31], disadvantaged backgrounds [33], or within a context of a parent-child agreement [32] and therefore provide limited generalizability to broader adolescent populations. Of the two studies conducted on general adolescent populations, one study exploring response shift in quality of life for healthy adolescents in England found significant decreases in physical health and significant increases in self-perception, mood and emotion, and bullying from pre-to-post-test [22]. The second study examined changes in HRQoL in Spanish children and adolescents and found that HRQoL decreased in eight out of 10 of the KIDSCREEN dimensions (including physical and psychological well-being, autonomy and parent relations, social support and peers, and school environment), and these changes were more notable in females than males and in older (13–17 years old) participants [23].

Therefore much of the research in this area is based on specific adolescent populations, with limited longitudinal studies conducted, and none in general Australian adolescent populations. The current study therefore aims to further examine HRQoL in a sample of secondary school-aged Australian adolescents over a three-year period in order to determine if HRQoL changes over time and if so, whether those changes differ across gender and age. As low levels of HRQoL may indicate an underlying physical and/or psychological health concern, these findings will be important in identifying what proportion of students could be at risk of developing health problems.

## Methods

### Ethics, consent, and permissions

The study was approved by the Western Sydney University Human Ethics Committee (approval number: H8715) and the Department of Education, New South Wales Ethics Committee, and was conducted according to the Helsinki ethical principles of research. All participants, and their parents, provided informed consent prior to their participation in the research, with an understanding that its findings would be published.

### Participants and setting

This paper reports on part of a broader study investigating the impact of community-identified interventions on social capital, and psychosocial and socioeconomic outcomes. Student participants (*n* = 403) were recruited from multicultural comprehensive government high schools (*n* = 6) in the Sydney metropolitan (*n* = 5) and regional (*n* = 1) communities of New South Wales, Australia. Four of these six schools were single-sex (two male, two female) and the other two schools were co-educational. These communities were selected based on the following criteria: the geographical location is one of reported economic and social disadvantage; an indication that relevant schools and the community would participate in this longitudinal study; and the industry partner (The Benevolent Society) having interest and presence in the area. Data at baseline (Time 1) was collected across academic years seven to nine and across three time periods. Across Time 1, 2, and 3 participants were aged 12–15, 13–16, and 14–17 years old respectively. In the seventh grade students’ transition from primary to secondary school, with grades seven, eight, and nine classified as junior secondary school.

### Data collection

Participants completed a pen and paper self-report questionnaire. The questionnaires were completed at each school with teaching and research staff present to ensure a familiar and consistent environment, and appropriately timed so not to interfere with school activities. Initially 1,933 eligible participants were contacted, 1,379 of which provided consent (response rate of 71 %). Of those, 1,111 completed the questionnaire at Time 1, 804 students completed the questionnaire at Time 2, and 525 students at Time 3. The response rate across the three time periods were 57 % at Time 1, 42 % at Time 2, and 27 % at Time 3. A total of 421 students completed the questionnaire across all time points, leading to an overall response rate of 22 %. The response rate over time is indicative of: consent requirement from both parents and students; students' presence and availability on the data collection day and time (and one follow up day for each data collection point); students changing school or leaving at the end of year 10; and opt out option if/when students chose to. Data from the first wave was collected in 2011 (Time 1) when the participants were in years 7–9. The second wave of data was collected in 2012 (Time 2), and the third wave was in 2013 when the participants were in years 9, 10, and 11 (Time 3).

### Measurement

In addition to questions pertaining to demographic information, the KIDSCREEN-27 instrument was used to measure HRQoL. The KIDSCREEN is not designed as a clinical diagnostic tool for screening psychiatric disorders, but rather, to measure overall well-being (HRQoL). The instrument contains 27 items and covers five dimensions:

1. Physical Well-being (5 items): explores the level of physical activity, energy and fitness of the child/adolescent and to what extent they feel unwell or complain of poor health.
2. Psychological Well-being (7 items): examines elements of psychological well-being, such as positive emotions and satisfaction with life, and the absence of loneliness and sadness.
3. Autonomy and Parents Relations (7 items): examines the quality of child/adolescent and parent/care giver interactions and the extent to which the child/adolescents feels loved and supported by the family. Furthermore, it explores level of autonomy that the child/adolescent perceives to possess, and the quality of financial resources perceived by the child/adolescent.
4. Social Support and Peers (4 items): explores the quality of the child’s/adolescents’ social relations and interactions with friends and peers, and the extent of their perceived support.
5. School Environment (4 items): explores the perceptions that a child/adolescents holds regarding their cognitive capacity, learning and concentration in the school environment. This dimension also examines how the child/adolescent views the relationship between themselves and their teachers [20].

The response range for the KIDSCREEN-27 is based on a 5-point Likert scale from ‘0’ – (*never/not at all*) to ‘5’ (*always*). The internal consistency values (Cronbach’s Alpha) of the self-report KIDSCREEN-27 are reported to be satisfactory across all five dimensions: Physical Well-being (.80); Psychological Well-being (.84); Autonomy and Parents Relations (.81); Social Support and Peers (.81) and School Environment (.81) and the test-retest reliability ranges from .61 to .74 [20].

### Statistical analyses

The self**-**report algorithm detailed in the KIDSCREEN Manual [20] was used to convert the total raw scores from each KIDSCREEN dimension into Rasch scores, and then convert the Rasch scores into t**-**values. The resulting t**-**values can then be used to make comparisons to international t**-**values based on 14 European countries. These values are normed to a mean of 50 and a standard deviation of 10 [20]. A series of one-way repeated measures analysis of variances (ANOVAs) were conducted to compare scores on each of the five KIDSCREEN dimensions across the three time periods, with each KIDSCREEN dimension analysed separately. Furthermore, a series of mixed between-within subjects ANOVAs were conducted in order to examine any gender or age differences across the time periods. All analyses were conducted using IMB SPSS Statistics (22.0), with statistical significance set at *p* < .05 (two-tailed). A power analysis using the G*Power 3 computer program [34] determined that a total sample size of 114 subjects was required to detect moderate effects (0.13) [35] with 95 % power when using a repeated measures ANOVA. According to Cohen’s [35] specifications for ANOVA analyses small, medium, and large effect sizes are classified as 0.02, 0.13, and 0.26 respectively.

For the purposes of reporting the analyses, the participants will be referred to as: Group 1 (comprising students who were in Year 7, 8, and 9 across T1, T2, and T3 respectively, aged 12–15 years); Group 2 (comprising students who were in Year 8, 9, and 10 across T1, T2, and T3 respectively, aged 14–16 years); and Group 3 (comprising students who were in Year 9, 10, and 11 across T1, T2, and T3 respectively, aged 15–17 years).

## Results

### Descriptive data

Student participants attended comprehensive government secondary schools in multicultural regions of Greater Western Sydney, and regional areas of New South Wales, Australia. After screening the data, four participants were excluded from the dataset as they were missing 50 % of data points at Time 1 (*n =* 3), 50 % of data points at Time 2 and 30 % from Time 3 (*n =* 1), meaning that t-values could not be derived and included in the analysis. Furthermore, screening for outliers identified 14 participants with inconsistent patterns of responding or spurious data and the decision was made to exclude them from the dataset. This led to a total sample size of 403 students (56 % female; mean age 13.3 years, *SD* = 0.86) who completed the questionnaire at Time 1, 2, and 3. Sixty-eight percent of participants spoke a second language at home; with Arabic being the most commonly spoken language (60 %) followed by other (non-specified) languages (12 %) and Vietnamese (9 %). The remaining (19 %) languages included Italian, Greek, Cantonese, Mandarin Chinese, Turkish, Persian, and Pacific Islander.

When participants did not complete at least one question across any of the dimensions, a Rasch score and subsequent T-value could not be calculated for that participant on the dimension which was missing the data (e.g. if a participant did not answer a question on the physical well-being dimension, then a T-value for the physical well-being dimension could not be calculated). Therefore this made the total sample size across each dimension variable. Consequently, sample size across analyses ranged from 359 to 403 participants. A comparison across the KIDSCREEN dimensions between the participants who completed all three time periods and those who did not, revealed statistically significant differences on two dimensions: social support and peers (*t*(1072) *=* -2.80, *p* = .005) and school environment (*t*(1075) *=* -3.47, *p* = .001). Students who did not participate at Time 2 and Time 3 scored lower on those two dimensions than students who did, however these differences were statistically small (*d =* 0.18 and *d =* 0.22 respectively). There were no significant differences between the gender, and age/grade of those who completed all three time periods, and those who did not.

### HRQoL across three time points

A series of one-way repeated measures ANOVAs with Greenhouse-Geisser corrections were conducted to compare scores on each KIDSCREEN dimension at Time 1, Time 2, and Time 3. The means, standard deviations, and dimensions are presented in Table 1. Results revealed no significant differences between time points on the physical wellbeing, psychological wellbeing, autonomy and parent relations, and school environment dimensions. The only significant difference found was for the social support and peers dimension (*F*(1.940) = 3.210, *p* = .041, ƞ_p_^2^ = .008), however, the difference was not significant enough to be detected by post hoc tests. Compared to European KIDSCREEN norms (Table 1), participants in this longitudinal study reported considerably lower levels of psychological wellbeing.

**Table 1:** Means and standard deviations from the repeated measures ANOVA for Time 1, Time 2, and Time 3 (*n* = 403)

KIDSCREEN dimensions	Statistics	Time 1	Time 2	Time 3	European norms^a^
Physical Well-being	Mean	46.58	46.33	46.33	48.57
	SD	7.78	8.57	8.19	
Psychological Well-being	Mean	39.27	38.68	38.80	48.83
	SD	6.72	5.94	5.98	
Autonomy and Parent Relations	Mean	49.32	50.42	50.01	49.41
	SD	12.46	13.66	12.94	
Social Support and Peers	Mean	52.47	52.03	50.75	49.62
	SD	10.98	12.17	11.74	
School Environment	Mean	50.91	51.77	50.12	48.44
	SD	11.06	11.39	11.86	

^a^ These norms are based on the KIDSCREEN European normative samples for male and female adolescents aged 12–18 years [18]

### Gender comparison on HRQoL across three time points

A series of mixed between-within subjects ANOVAs were conducted to determine if there were gender differences in KIDSCREEN scores across the three time points, while controlling for grade/age. ANOVA assumptions were satisfactory, with the exception of Mauchley’s test of sphericity, which was significant across all dimensions and therefore Wilks’ Lambda results were reported [36]. Means standard deviations, and dimensions are presented in Table 2.

**Table 2:** Means and standard deviations from the mixed between-within subjects ANOVA for Time 1, Time 2, and Time 3 (*n* = 403)

KIDSCREEN dimensions	Time 1 Mean (SD)	Time 2 Mean (SD)	Time 3 Mean (SD)
Physical Well-being			
Female	45.01 (7.08)^a^	45.21 (8.17)^a^	44.31 (7.78)^a^
Male	48.59 (8.20)^a^	47.17 (8.77)^a,b^	48.93 (8.00)^a^
Group 1	48.69 (7.50)^d^	47.44 (8.47)^d^	48.17 (8.07)^d^
Group 2	44.91 (8.00)	44.89 (8.62)	45.06 (8.25)
Group 3	43.93 (5.86)	44.78 (8.09)	43.36 (6.74)
Psychological Well-being			
Female	38.73 (4.90)^a^	38.41 (4.96)^a^	37.75 (4.60)^a^
Male	39.97 (8.47)^a^	39.03 7.01)^a^	40.15 (7.17)^a^
Group 1	39.94 (6.56)^d^	39.49 (6.44)^d^	39.52 (6.64)^d^
Group 2	38.76 (7.43)	37.93 (5.75)	38.42 (5.50)
Group 3	38.45 (3.61)	38.20 (3.73)	37.31 (4.25)
Autonomy and Parent Relations			
Female	47.90 (12.26)^a^	49.17 (14.06)^a^	47.49 (11.85)^a,c^
Male	51.15 (12.26)^a^	52.03 (14.06)^a^	53.25 (13.58)^a^
Group 1	50.26 (12.06)	51.79 (13.24)	52.39 (12.96)
Group 2	48.99 (13.38)	49.81 (15.02)	48.57 (13.11)
Group 3	47.07 (10.21)	47.57 (8.97)	46.43 (10.71)
Social Support and Peers			
Female	52.78 (10.11)	52.86 (11.14)	50.91 (10.50)^c^
Male	52.06 (12.04)	50.96 (13.34)	50.55 (13.20)
Group 1	53.49 (11.25)^d^	53.09 (11.87)^d^	52.71 (11.28)^d^
Group 2	51.60 (11.13)	51.05 (12.97)	48.59 (12.45)
Group 3	51.47 (9.09)	51.26 (10.22)	50.57 (9.65)
School Environment			
Female	50.36 (10.70)	52.08 (10.56)	49.37 (11.05)^c^
Male	51.61 (11.50)	51.36 (12.40)	51.07 (12.80)
Group 1	52.93 (11.14)^4^	52.79 (11.06)^d^	52.32 (11.87)^d^
Group 2	48.74 (10.49)	50.67 (12.24)	47.57 (12.24)
Group 3	50.13 (11.37)	51.32 (9.41)	49.91 (8.42)

^a^Significant main effect for gender

^b^ Significantly lower scores at Time 2 compared to Time 3

^c^ Significantly lower scores at Time 3 compared to Time 2

^d^Significant main effect for grade/age

There were significant ordinal interactions between gender and time on two of five dimensions: the physical wellbeing (Wilks’ Lambda = .980, *F*(2, 355) = 3.682, *p =* .026, ƞ_p_^2^ = .020) and psychological wellbeing (Wilks’ Lambda = .983, *F*(2, 368) = 3.133, *p =* .045, ƞ_p_^2^ = .017) dimensions, with males reporting significantly higher scores than females across these dimensions. The effect sizes were, however, small.

There were no significant main effects for time across any of the KIDSCREEN dimensions. That is, time did not uniquely contribute to changes in KIDSCREEN scores for the total sample. However, the main effect for gender was significant across three of the five dimensions: the physical wellbeing (*F*(1, 356) = 19.061, *p <* .001, ƞ_p_^2^ = 0.051), psychological wellbeing (*F*(1, 369) = 6.865, *p =* .009, ƞ_p_^2^ = 0.018), and autonomy and parent relations (*F*(1, 367) = 10.163, *p* = .002, ƞ_p_^2^ = 0.027), with males scoring significantly higher than females on each of the three dimensions. Despite these significant findings, the effect sizes were small.

Post hoc analyses revealed that for males, there was a significant shift over time in scores on the physical wellbeing dimension, *F*(1.982, 309.226) = 3.235, *p* = .041, ηp^2^ = 0.020, although the effect size was small. Males reported significantly lower scores at Time 2, compared to Time 3. There were no significant changes across the other dimensions.

For females, there were significant shifts across time periods on two of the five dimensions: the social support and peers (*F*(1.838, 406.228) = 3.188, *p* = .046, ηp^2^ = 0.014), and school environment (*F*(1.723, 379.024) = 5.014, *p* = .010, ηp^2^ = 0.022) dimensions. Females reported significantly lower scores at Time 3, compared with Time 2 on both dimensions; however these effect sizes were small.

### Grade/age comparison on HRQoL across three time points

In regards to grade/age, when controlling for gender, there were no significant time by grade interactions (Wilks’ Lambda’s ≥ .988, *F*s ≥ .136, *p*s ≥ .302, ƞ_p_^2^ ≥ .001), or main effects for time (Wilks’ Lambda’s ≥ .984, *F*s ≥ .490, *p*s ≥ .054, ƞ_p_^2^ ≥ .003). The main effect for grade/age was significant across four of five dimensions: the physical wellbeing (*F*(2, 355) = 23.245, *p* < .001, ƞ_p_^2^ = .061), psychological wellbeing (*F*(2, 368) = 8.265, *p* = .004, ƞ_p_^2^ = .022), social support and peers (*F*(2, 389) = 5.171, *p* = .006, ƞ_p_^2^ = .026), and school environment (*F*(2, 390) = 8.744, *p* < .001, ƞ_p_^2^ = .043) dimensions. Students in Group 1 reported significantly higher scores than students in Group 2 across the three time periods on these dimensions, however these effects were small. While these were the only significant declines, the other age groups also reported declines in social support and peers scores over time (but not at statistically significant levels).

## Discussion

The aim of this study was to explore HRQoL over a period of three years in a sample of school-based adolescents and to examine if there were gender and age differences. This sample of Australian students reported similar levels of HRQoL as European adolescents with the exception of lower levels on psychological wellbeing dimension. The overall sample fluctuated on school environment dimension, which was higher at Time 2 than Time 1 or 3 while social support and peers declined across three time points.

However, on closer examination, it appears those changes over time were gender related. That is, gender differences were found across physical and psychological wellbeing and autonomy and parent relations, with males reporting higher levels than females across all three dimensions. Males had a significant decline in physical wellbeing at Time 2 but seem to ‘bounce’ back at Time 3. Females, however, had a steady decline in psychological wellbeing over three time points and a decline across all five dimensions between Time 2 and Time 3. These differences suggest gender specific trajectories, which future studies could explore with mixture modelling to identify if they are normally distributed or if there are sub-gender and age patterns.

When examining age differences, there was a notable decline in scores on social support and peers across all age groups over time, although only some were statistically significant. Students in Group 1 reported significantly higher scores than students in Group 2 across the three time periods on the physical and psychological well-being, social support and peers, and school environment dimensions. As other declines were not statistically significant, these findings are consistent with previous research reporting that HRQoL remains relatively stable in older adolescents [23].

Some of the findings in this study however confirm those of studies that have reported HRQoL to decline over time and for this pattern to be more evident for females than males [24–26]. In this study, for females the decline was significant across two of five dimensions from Time 2 to Time 3, while remaining relatively stable, if albeit significantly lower than males’, for physical and psychological well-being, and autonomy and parent relations. Although those findings are statistically significant, the effect sizes were predominantly small, which suggests that the differences would not be readily noticeable nor would be clinically meaningful. It is possible that the conceptualisation of some domains may change over time with a shift in the internal standards used to self-evaluate quality of life, which may explain some of the age differences [29]. This shift was also reported in a study that examined parent-child corroboration of quality of life and found that a gap in perceptions widens with adolescents’ age [32]. The pattern of gender differences (i.e. quality of life being lower for females and declining over time), may be highlighting a gender-specific vulnerability during adolescence. Given that those changes are not readily identifiable, fostering awareness and preventative measures may protect from further loss of HRQoL.

This study is not without limitations. These include: (1) a narrow geographic, economic and multi-cultural focus which, while sampling school-based adolescents, may not be representative of broader adolescent population; (2) participants’ self-selection and retention over time, may also contribute to a representation bias; (3) the collection of self-report data may have had an impact on the results (see discussion below); (4) no information was gathered on potential events and stressors that may have influenced participants’ responses across HRQoL dimensions and time; (5) lastly, no clinical assessment was included to investigate if variations over time and sub-groups may be related to participants’ mental and physical health problems. The latter two points are recommended as areas of future research.

In regards to the collection of self-report data, while some research has raised concerns regarding the capacity of children and adolescents to adequately report their experience of psychological and physical health problems [37], others have shown that adolescents completing self-report measures were slightly better at predicting health outcomes than their parents [38]. Furthermore, factors such as social desirability and comparisons to others [39], as well as health concerns [40] may affect adolescents’ self-perception and their responses on the KIDSCREEN questionnaire. However, those possible limitations are common across studies and do not prevent comparisons or generalizability of findings.

Notwithstanding those limitations, this study provides an informative longitudinal snapshot of HRQoL in a school-based adolescent population and confirms gender and age differences that appear over time. Future research may build on these findings by focusing on the age points when gender differences start to emerge to better understand what may be influencing those HRQoL changes.

## Conclusions

This longitudinal study found that HRQoL changes over time and seems to be predominantly gender influenced, with females reporting somewhat poorer QoL than males. Those differences may be discrete but nonetheless indicative of potential vulnerability during this stage of development. Given the risk of psychological/mental and physical health problems that emerge in adolescents potentially continuing into adulthood, identifying and addressing early signs of HRQoL decline provides an opportunity to ensure that young people have a healthier progression through adolescence.
